# Using a new human milk fortifier to optimize human milk feeding among very preterm and/or very low birth weight infants: a multicenter study in China

**DOI:** 10.1186/s12887-024-04527-2

**Published:** 2024-01-19

**Authors:** Junyan Han, Lan Zhang, Rong Zhang, Shuping Han, Jianxing Zhu, Xuefeng Hu, Jianhua Sun, Gang Qiu, Zhenghong Li, Weili Yan, Lijuan Xie, Xiuxia Ye, Xiaohui Gong, Liling Li, Fei Bei, Chan Liu, Yun Cao

**Affiliations:** 1 Department of Neonatology, Children’s Hospital of Fudan University, National Children’s Medical Center, 399 Wanyuan Road, Minhang District, Shanghai, 201102 China; 2 Department of Pediatrics, Nanjing Maternity and Child Health Care Hospital, Women’s Hospital of Nanjing Medical University, Nanjing, Jiangsu Province 210004 China; 3 Department of Neonatology, Xinhua Hospital, Shanghai Jiaotong University School of Medicine, Shanghai, 200092 China; 4 Department of Neonatology, Shanghai First Maternity and Infant Hospital, School of Medicine, Tongji University, Shanghai, 200092 China; 5 Department of Neonatology, Shanghai Children’s Medical Center, School of Medicine, Shanghai Jiao Tong University, Shanghai, 200127 China; 6 Department of Neonatology, Shanghai Children’s Hospital, School of Medicine, Shanghai Jiao Tong University, Shanghai, 200040 China; 7 Department of Pediatrics, Peking Union Medical College Hospital, Chinese Academy of Medical Science and Peking Union Medical College, Beijing, 100730 China; 8 Department of Clinical Epidemiology, Children’s Hospital of Fudan University, National Children’s Medical Center, Shanghai, 201102 China

**Keywords:** Feeding intolerance, Growth, Human milk fortifier, Infant

## Abstract

**Background:**

Human milk fortifier (HMF) composition has been optimized recently. But clinical evidence of its safety and efficacy is limited in Chinese population. The aim of this study was to evaluate effects of a new HMF in growth, nutritional status, feeding intolerance, and major morbidities among very preterm (VPT) or very low birth weight (VLBW) infants in China.

**Methods:**

VPT/VLBW infants admitted from March 2020 to April 2021 were prospectively included in the experimental (new HMF, nHMF) group, who received a new powdered HMF as a breast milk feeding supplement during hospitalization. Infants in the control group (cHMF) admitted from January 2018 to December 2019, were retrospective included, and matched with nHMF group infants for gestational age and birth weight. They received other kinds of commercially available HMFs. Weight gain velocity, concentrations of nutritional biomarkers, incidence of major morbidities, and measures of feeding intolerance were compared between the two groups.

**Results:**

Demographic and clinical characteristics of infants in nHMF and cHMF groups were comparable. Weight gain velocity had no significant difference between the nHMF (14.0 ± 3.5 g/kg/d) and the cHMF group (14.2 ± 3.8 g/kg/d; *P* = 0.46). Incidence of morbidities, including necrotizing enterocolitis, bronchopulmonary dysplasia, retinopathy of prematurity, culture-confirmed sepsis, and feeding intolerance during hospitalization between nHMF and cHMF, were similar (all *P*-values > 0.05). The time to achieve full enteral feeding [13.5 (10, 21) days] in the nHMF group was significantly shorter than that in the cHMF group [17 (12, 23) days, HR = 0.67, 95%CI: 0.49, 0.92; *P* = 0.01]. Compared with cHMF group, the decrease of blood urea nitrogen level over time in nHMF group was smaller (β = 0.6, 95%CI:0.1, 1.0; *P* = 0.01).

**Conclusions:**

The new HMF can promote growth of preterm infants effectively without increasing the incidence of major morbidity and feeding intolerance. It can be used feasible in Chinese VPT/VLBW infants.

**Trial registration:**

This study was registered on ClinicalTrials.gov (NCT04283799).

**Supplementary Information:**

The online version contains supplementary material available at 10.1186/s12887-024-04527-2.

## Background

According to the World Health Organization, approximately 10.6%, or around 14.84 million births worldwide were preterm in 2014, around 1.17 million preterm births occurred annually in China, ranking second in the world [1]. Preterm infants were at high risks of extrauterine growth restriction (EUGR) and long-term complications. The smaller the gestational age and the lower of the birthweight, the greater the risk of EUGR [2, 3].

Compared to formula feeding, human milk (HM) provides numerous benefits for preterm infants, including improved nutritional, immunological, and metabolic outcomes. Furthermore, HM feeding is not only associated with lower incidence of necrotizing enterocolitis (NEC), sepsis, bronchopulmonary dysplasia (BPD), and retinopathy of prematurity (ROP) during early life, but also related with lower risk of asthma, inflammatory bowel disease, autoimmune diseases and metabolic diseases in later life [4, 5]. However, exclusive HM feeding often fails to provide sufficient protein and micronutrients for the rapid growth demands of preterm infants, especially in very preterm infants. Human milk fortifier (HMF) was developed to supplement HM nutrient composition and was recommended for use in preterm infants with birth weight of less than 1800 g [6]. Fortified HM can significantly improve protein and total energy intake, which is conducive to promoting the in-hospital growth rate of preterm infants [7].

Based on further examination of the nutritional requirements of preterm infants, a new powdered HMF with optimized composition was recently developed, including higher content of protein, calories, protein-to-energy ratio, and medium-chain fatty acids, and lower osmotic pressure [8]. The new HMF is made entirely from partially hydrolyzed whey protein (Supplementary Table 1). Mixed with HM, it delivers approximately about 3.6 g/100 kcal protein and has an osmolality of 339 mOsm/L, meeting the recommendations of the European Society for Pediatric Gastroenterology Hepatology and Nutrition (ESPGHAN) [9].

A clinical trial by Rigo, et al. [8] demonstrated that the new HMF could provide higher levels of protein and lipids, and it was safe, well-tolerated, and could improve the weight gain of preterm infants. However, in Chinese population, the feeding strategy and the kind of control HMFs were different from the previous study. In addition, clinical evidence on effects of new HMF on other short-term outcomes of preterm infants, such as NEC, ROP and BPD, was limited. Therefore, the aim of this multicenter study was to evaluate differences in growth, nutritional status, feeding intolerance, and major morbidity (NEC, BPD, ROP, and sepsis) between the new HMF and previous HMFs in a population of very preterm (VPT, with gestational age over 28 weeks and less than 32 weeks) and very low birth weight (VLBW, with birth weight over 1000 g and less than 1500 g) infants in China.

## Methods

### Study design and participants

This multicenter, single-arm study with a historical control group was a non-inferiority study, conducted in neonatal intensive care units (NICUs) of seven children’s hospitals and maternal and child health hospitals across Shanghai, Beijing, and Nanjing, China. The study was reviewed and approved by the Institutional Ethics Committees of each study site.

The prospectively enrolled experimental group (nHMF) was comprised of infants admitted to the study NICUs from March 2020 to April 2021. These infants received the new HMF, and met all of the study inclusion criteria: (1) birth weight of 1000-1499 g and/or gestational age between 28 + 0 and 31 + 6 weeks; (2) received over 50% of their total enteral feeding volume as human milk (HM) during hospitalization, including mother’s own or donor milk; (3) were either born in or transferred to study centers within 24 h after birth; (4) in case of twins or multiple births, only the first-born was included; (5) informed consent was obtained from the parents/guardians of eligible infants. We excluded infants who met one of the following criteria: (1) with major congenital anomalies, severe asphyxia, or severe intracranial hemorrhage; (2) small for gestational age (SGA, birth weight < 10th percentile of the Fenton reference) [10]; (3) participating in other clinical trials.

The retrospective control group (cHMF) was comprised of preterm infants admitted to the abovementioned NICUs between January 2018 and December 2019, a period when the new HMF was not available in the Chinese market. Each infant in the nHMF group should be matched with two infants in control group based on gestational age and birth weight with differences less han one week and 100 g, respectively. The inclusion criteria for the control group mirrored those of the nHMF group. In addition, these infants also met the following criteria: (1) received HMF consecutively for at least 21 days post full fortification during hospitalization; (2) completed body weight measurements at least thrice weekly during hospitalization. The exclusion criteria were identical to those of the nHMF group.

### Study procedure

#### Feeding strategy and HMF usage

Across all study sites and for both groups, the feeding strategies and HMF usage were standardized. Enteral feeding was generally initiated within 24 h after birth, except in cases where the infant presented high risks of congenital gastrointestinal malformations or serious circulatory issues. Mother’s own milk was the preferred primary feeding source. If the mother’s own milk was insufficient or unavailable, donor milk was preferred. Feeding was administered intermittently every 2 or 3 h using either a nasogastric or orogastric tube. The rate of feeding advancement was 10-40mL/kg/day determined by the attending physicians based on the infant’s clinical status. All infants had been transferred from tube feeding to oral feeding before discharge.

HMF using started (Day0, D0) when infants tolerated ≥ 80 mL/kg/day of enteral feeding for at least 24 h. The dosage of HMF was gradually increased from 1 g to 0.9 g HMF/100mL HM to full-strength fortification according to the different product manual and clinical condition within five to seven days. The day when full-strength fortification first achieved was designated as Day 1 (D1). Each infant was required to receive full-strength fortification for at least 21 days (until Day 21, D21). Infants continued to receive their study HMF until NICU discharge or medical decision to stop fortification. Before each feeding, HMF was thoroughly mixed with HM at the bedsides and administered to infants immediately (within 15 min). The HMF used in the nHMF group was preNAN Human Milk Fortifier (Nestlé, Switzerland), and the HMF used in cHMF group consisted of commercially available fortifiers used during the study period. The nutritional content of each HMF is listed in Supplementary Table 1.

#### Growth

Infants nude weight (accurate to 1 g) was measured three times per week during the study period. A standardized physical measurement method and the same equipment (SECA 376 electronic baby scale, Hamburg, Germany) were used in each study center. Both weight gain velocity and change of weight z-score were used to evaluate growth velocity in this study. Weight gain velocity was calculated using the exponential model: GV (g/kg/d) =[1000*ln(Wn/W1)]/(Dn-D1) [11]. Weight-for-age z-scores were computed using the Lambda-Mu-Sigma (LMS) parameters based on the Fenton growth chart (2013) [10]. The primary outcome of this study was weight gain velocity from D1 to D21. Furthermore, weight gain velocity from D1 to the 14th day of full-strength fortification (D14) and from D1 to discharge were also compared between the two groups.

#### Feeding tolerance

Feeding intolerance [12, 13] was diagnosed when two of the following three criteria were met: (1) vomiting ≥ 3 times/day; (2) gastric residual volume > 50% of previous feeding volume ≥ 3 times/day or gastric residual volume > 33% of previous feeding volume ≥ 5 times/day; (3) abdominal distension and hypoactive bowel sounds. The time to achieve full enteral feeding (enteral feeding volume ≥ 120 ml/kg/day) was also recorded in this study.

#### Markers of nutritional status

Blood samples were collected at D0 and D21, and analyzed for serum prealbumin, albumin, sodium, blood urea nitrogen (BUN), hemoglobin, calcium, phosphorus, total cholesterol, triglycerides, and alkaline phosphatase. All blood parameters were analyzed as part of routine clinical assessments at each study center.

#### Safety and adverse events

During the study period, frequency, type, and attribution to HMF intake of adverse events (AEs) were evaluated by study physicians using a standardized form. Incidence of major morbidities, including NEC, BPD, ROP, and sepsis, were collected from medical charts for each infant. NEC was defined as greater than or equal to stage II according to the Modified Bell’s criteria [14]. Infants who required oxygen therapy > 21% for at least 28 days were classified as having BPD at 36 weeks postmenstrual age using National Institutes of Health (NIH) consensus definition [15]. Diagnosis of ROP and staging were categorized according to international classification for ROP [16]. Sepsis in this study refers to sepsis confirmed by blood culture [17].

### Ethical considerations

This study was reviewed and approved by the Institutional Ethics committee of each research center. All parent(s)/guardian(s) of each participant in the nHMF group understood the research plan and objective, and voluntarily signed an informed consent form before participating. Data of participants in the cHMF group were collected retrospectively from medical charts without personal identification information. This study was prospectively registered on ClinicalTrials.gov (NCT04283799) and the initial release date was 23/02/2020.

### Sample size calculation

The sample size was calculated using PASS 16 Power Analysis and Sample Size Software (NCSS, LLC. Kaysville, Utah, USA) based on the primary hypothesis that weight gain velocity of the experimental group would be faster than the control group. According to Rigo et al. [8], the weight gain in experimental group and control group were assumed to be 18.3 ± 3.7 g/kg/d and 16.8 ± 3.7 g/kg/d. The ratio of infant number in control group and nHMF group was two to one. A total of 73 infants in nHMF group and 146 infants in cHMF group are needed to achieve 80% power with a significance level of 0.05 using a two-sided two sample equal-variance t-test. Considering a dropout rate of 20% of nHMF group, we planned to recruit 92 infants in nHMF group.

### Statistical analyses

Mean and standard deviation (SD), or median and inter-quartile range (IQR) were used to describe continuous variables according to the distribution of data. N (%) was used to describe categorical variables. Given all of the growth parameters were continuous variables and the matched-pair study, linear mixed effect models were used to compare the difference of growth parameters between nHMF and cHMF group. The nHMF and cHMF groups were as fixed effect, and matched pair was as random effect in these models. Gestational age, birthweight, age of start using HMF, and days to achieve full enteral feeding were adjusted as covariates. Adjusted mean differences and a 95% confidence interval (CI) were reported. Given that body weight and blood biomarkers were repeated measurements (two times for each infant), generalized estimating equations (GEE) models were performed to describe the changes from D0 to D21, using an identity link function and an exchangeable correlation matrix assuming Gaussian distribution for blood marker levels. Gestational age, sex, birth weight, age at start of HMF use (D0), days to achieve full enteral feeding, and study site were adjusted as covariates in GEE models. Multiple logistic regression models were used to compare the difference of feeding intolerance incidences and morbidities between the two groups. Cox regression models were used to compare the difference of time to achieve full enteral feeding and time to full-strength fortification. Gestational age, sex, birth weight, age at start of HMF usage were adjusted as covariates, odds ratio (OR) and hazard ratio (HR) values with 95%CI were reported. *P* < 0.05 was considered statistically significant. Stata 15.0(Stata Corp, College Station, TX, USA)was used for all statistical analyses.

## Results

### Clinical characteristics

**Fig. 1:**
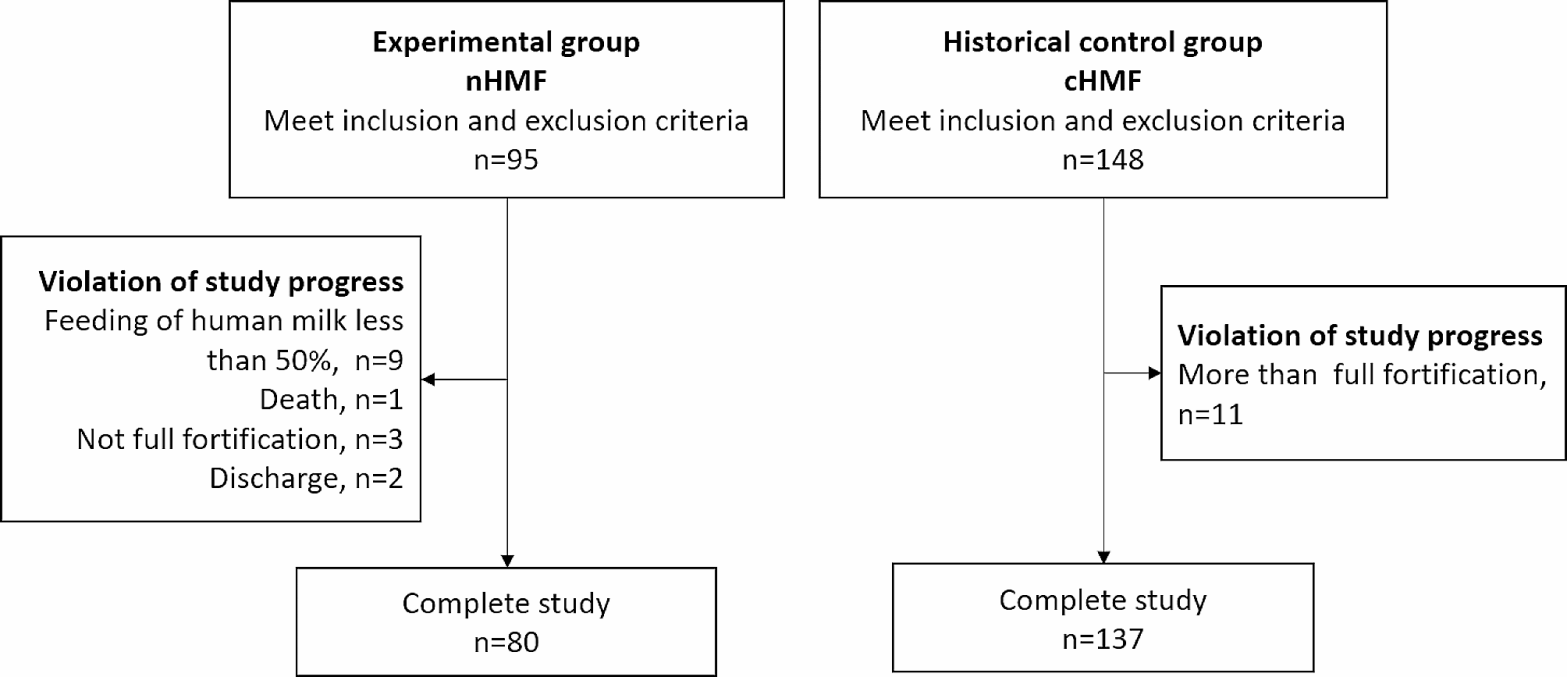
Flow chart of preterm infants. nHMF, new human milk fortifier; cHMF, control human milk fortifier

According to the inclusion and exclusion criteria, a total of 95 infants were recruited into the nHMF group. In the cHMF group, we included 148 infants whose gestational age and birth weight were matched with those in nHMF group. In other words, 53 of 95 cases in nHMF group only found one matched case. Figure 1 showed the flow chart of this study. During the study period, 15 infants in the nHMF group did not complete the study for the following reasons: insufficient HM feeding (less than 50% of total feeding volume, *n* = 9), mortality (*n* = 1), failure to achieve full-strength fortification (*n* = 3), and discharge against medical advice (*n* = 2). In the cHMF group, 11 infants received over full-strength fortification HM feeding as deemed necessary by the attending physician. Consequently, 80 infants in nHMF and 137 infants in cHMF group completed the intervention, providing sufficient statistical power for testing our primary outcome.

The demographic and clinical characteristics of participants are shown in Table 1. Gestational age, birth weight, sex distribution, cesarean section rate, birth asphyxia, prenatal disease, time to full enteral feeding, and length of NICU stay were similar between the two groups of preterm infants (all *P* values > 0.05). Notably, the nHMF group began HMF administration earlier (11.9 ± 6.3 days) compared to the cHMF group (17.2 ± 8.9 days, *P* < 0.001).

**Table 1:** Characteristics of infants in the nHMF group and cHMF group

Characteristics	nHMF group (*n* = 95)	cHMF group (*n* = 148)	*P*-Value
**Neonatal characteristics**			
Gestational age, weeks	29.9 ± 1.2	29.7 ± 1.2	0.15
Birth weight, g	1338 ± 177	1354 ± 148	0.46
Birth weight z-score	0.06 ± 0.67	0.08 ± 0.68	0.86
Male, n (%)	48 (50.5)	72 (49.0)	0.69
Caesarean section, n (%)	59 (62.1)	84 (57.9)	0.68
Apgar score − 5 min	8.9 ± 1.1	9.0 ± 1.2	0.53
Age at start of HMF use, days	11.9 ± 6.3	17.2 ± 8.9	< 0.001
Time to achieve full-strength fortification, days	6.8 ± 5.6	6.9 ± 4.7	0.89
Length of NICU stay, days	52.2 ± 15.0	50.4 ± 14.1	0.24
**Maternal characteristics**			
Antenatal corticosteroid, n (%)	74 (77.9)	112(75.7)	0.76
Gestational hypertension, n (%)	17 (17.9)	19 (12.8)	0.35
Gestational diabetes mellitus, n (%)	19 (20.0)	32 (21.6)	> 0.99
Premature rupture of membrane, n (%)	31 (32.6)	40 (27.0)	0.24

Mean and standard deviation (SD) was used to describe continuous variables. Student *t* test were used to compare the differences between the two groups and Fisher exact test was used to compare the two groups for categorical variables

nHMF, new human milk fortifier; cHMF, control human milk fortifier

### Weight gain

Table 2 shows the weight gains of the two groups. At various stages of the study (D1, 14, 21, and at discharge), differences in both absolute weight and weight z-scores were not significant between the nHMF and cHMF groups. From D1 to D21, the absolute weight gain [(533 ± 165) g vs. (540 ± 173) g; *P* = 0.38], weight gain velocity [(14.0 ± 3.5) g/kg/d vs. (14.2 ± 3.8) g/kg/d; *P* = 0.46], and change of body weight-for-age z-score [(-0.28 ± 0.43) vs. (-0.20 ± 0.44); *P* = 0.23] in the nHMF and cHMF group were similar. However, a notable difference was observed in the absolute weight gain from D1 to the time of discharge, with the nHMF group exhibiting a higher gain (878 ± 473 g) compared to the cHMF group (730 ± 372 g; *P* = 0.01), resulting in a mean difference of 148 g. Despite this, the weight gain velocity and change of body weight z-score during that period did not significantly differ between the two groups (*P* = 0.40 and *P* = 0.09, respectively). Furthermore, no significant difference was observed in absolute weight gain (*P* = 0.93) and weight gain velocity (*P* = 0.78) from D1 to D14 across the two groups.

**Table 2:** Body weight and weight gain velocity of infants in the nHMF group and cHMF group^*^

Weight or weight gain	nHMF group (*n* = 95)	cHMF group (*n* = 148)	β (95%CI)^#^	*P*-Value
**Weight**				
D1, kg	1.53 ± 0.22	1.57 ± 0.26	-0.00(-0.05, 0.06)	0.88
D14, kg	1.86 ± 0.27	1.90 ± 0.33	-0.03(-0.05, 0.10)	0.49
D21, kg	2.04 ± 0.32	2.11 ± 0.36	-0.06(-0.03, 0.14)	0.19
Discharge, kg	2.39 ± 0.48	2.29 ± 0.45	0.11(-0.02, 0.24)	0.10
**Weight for age z-score**				
D1	-0.87 ± 0.63	-1.07 ± 0.74	0.01 (-0.14, 0.14)	0.31
D14	-1.10 ± 0.65	-1.28 ± 0.86	0.08(-0.08, 0.24)	0.12
D21	-1.16 ± 0.71	-1.28 ± 0.92	0.10(-0.09, 0.29)	0.29
Discharge	-1.20 ± 0.97	-1.24 ± 0.87	0.18(-0.08, 0.30)	0.56
**Weight gain from D1 to D21**				
Absolute weight gain, g	533 ± 165	540 ± 173	0.02(-0.03, 0.08)	0.38
Weight gain velocity, g/kg/d	14.0 ± 3.5	14.2 ± 3.8	0.42(-0.69, 1.52)	0.46
Change of z-score	-0.28 ± 0.43	-0.20 ± 0.44	0.09(-0.05, 0.22)	0.23
**Weight gain from D1 to discharge**				
Absolute weight gain, g	878 ± 473	730 ± 372	-0.17(-0.29, -0.03)	0.01
Weight gain velocity, g/kg/d	13.5 ± 4.5	14.3 ± 6.3	0.83 (-1.1, 2.6)	0.40
Chang of z-score	-0.32 ± 0.77	-0.18 ± 0.45	0.18 (-0.01, 0.35)	0.09
**Weight gain from D1 to D14**				
Absolute weight gain, g	338 ± 126	332 ± 137	0.01(-0.04, 0.04)	0.93
Weight gain velocity, g/kg/d	14.3 ± 4.6	13.6 ± 4.8	-0.20(-1.59, 1.20)	0.78
Change of z-score	-0.21 ± 0.33	-0.19 ± 0.36	0.03(-0.07, 0.13)	0.55

^*^ In the nHMF group, 2 infants were discharged before the end of the trial, we impute the body weight z-score at D21 as the same as the body weight z-score at discharge. Data was imputed using the mean of body weight z-score in nHMF group for the infant who died during study period

^#^ Linear mixed effect models were used to compare the difference of growth parameters between nHMF and cHMF group. Gestational age, birthweight, age of start using HMF, and days to achieve full enteral feeding were adjusted as covariates

D1, D14, D21, the 1st, 14th, and 21st day after full-strength fortification; nHMF, new human milk fortifier; cHMF, control human milk fortifier

### Markers of nutritional status

The levels of various nutritional biomarkers on D0 and D21 for both groups are summarized in Table 3. After adjusting for confounding factors, there were no significant difference in change of albumin, prealbumin, sodium, calcium, phosphorus, alkaline phosphatase, total cholesterol, and triglycerides levels between the nHMF and cHMF groups (all *P*-values > 0.05). However, the reduction of hemoglobin level of infants in the nHMF group was significantly greater than that of the cHMF group (β=-1.4, 95%CI: -2.3, -0.5; *P* = 0.004). In addition, the level of blood urea nitrogen (BUN) decreased less in the nHMF group than in the cHMF group (β = 0.6, 95%CI: 0.1,1.0; *P* = 0.01).

**Table 3:** Changes of nutritional biomarker levels of infants in nHMF and cHMF group from D0 to D21

Nutritional Indicator	nHMF Group (*n* = 76)			cHMF Group (*n* = 138)			β (95%CI) ^*^	*P*-Value
	D0	D21		D0	D21			
Hemoglobin, g/dL	14.3 ± 3.4	10.4 ± 2.6		12.4 ± 3.9	9.9 ± 3.2		-1.4 (-2.3, -0.5)	0.004
Albumin, g/L	32.1 ± 4.6	31.2 ± 3.1		30.8 ± 3.9	30.5 ± 3.7		-0.9 (-3.5, 0.5)	0.10
Prealbumin, mg/L	84.1 ± 28.7	83.7 ± 22.1		79.2 ± 32.3	67.8 ± 25.1		-2.4 (-11.2, 6.4)	0.59
Sodium, mmol/L	137 ± 3.5	137 ± 2.6		138 ± 3.1	138 ± 2.8		0.8 (-0.04, 1.7)	0.08
Calcium, mmol/L	2.35 ± 0.20	2.40 ± 0.16		2.32 ± 0.30	2.36 ± 0.22		-0.02 (-0.09,0.05)	0.56
Phosphorus, mmol/L	1.70 ± 0.48	2.02 ± 0.29		1.70 ± 0.42	1.98 ± 0.31		-0.06 (-0.16, 0.05)	0.28
Alkaline phosphatase, U/L	365 ± 128	312 ± 100		373 ± 155	335 ± 124		3.25(-33.3, 39.8)	0.86
BUN, mmol/L	3.18 ± 1.57	2.34 ± 0.93		3.39 ± 2.17	1.78 ± 1.18		0.6 (0.1, 1.0)	0.01
Total cholesterol, mmol/L	3.01 ± 0.89	2.90 ± 0.69		2.75 ± 0.75	2.63 ± 0.57		-0.12 (-0.32, 0.07)	0.21
Triglyceride, mmol/L	0.93 ± 0.48	0.78 ± 0.25		0.85 ± 0.47	0.90 ± 0.39		-0.06 (-0.18, 0.06)	0.34
25-hydroxyvitamin D, ng/mL	36.3 ± 13.1	40.8 ± 13.7		20.1 ± 8.2	25.7 ± 9.9		-4.09 (-18.7, 10.5)	0.58

A total of 76 infants in nHMF group and 138 infants in cHMF group completed the blood samples test twice

Mean and standard deviation (SD) was used to describe continuous variables; Infants who complete the study were included in these analyses

^*^ Gestational age, sex, birth weight, age of start using HMF, days to achieve full enteral feeding, and study site were justified in generalized estimating equation models. nHMF, new Human milk fortifier; cHMF, control human milk fortifier; BUN, blood urea nitrogen; D0, start using HMF, D21, the 21st day of full-strength fortification

### Feeding Tolerance

The feeding intolerance incidence in the nHMF group was comparable to that of the cHMF group (OR = 1.12, 95%CI: 0.26, 4.8; *P* = 0.87, as shown in Table 4). The nHMF group reached full enteral feeding significantly faster than cHMF group (HR = 0.67, 95%CI: 0.49, 0.92; *P* = 0.01). However, there was no significant difference in the time to achieve full-strength fortification between the two groups (HR = 1.12, 95%CI: 0.84, 1.49; *P* = 0.46).

**Table 4:** Feeding tolerance and morbidities during hospitalization in the nHMF and cHMF group

Feeding tolerance and morbidity	nHMF group (*n* = 95)	cHMF group (*n* = 148)	OR/HR (95%CI)	*P*-Value
Feeding intolerance, n (%)	5 (5.2)	8 (5.4)	1.12(0.26, 4.8)	0.87
Necrotizing enterocolitis ≥ 2 stage, n (%)	1(1.05)	2(1.35)	0.67(0.04, 10.4)	0.77
Bronchopulmonary dysplasia^*^, n (%)	30(31.9)	61(41.2)	0.88(0.44, 1.78)	0.73
Retinopathy of prematurity ≥ 2 stage, n (%)	10(13.6)	37(25.0)	2.10 (0.92, 4.76)	0.07
Culture-confirmed sepsis, n (%)	3(3.1)	7(4.7)	2.38(0.76, 6.32)	0.14
Time to achieve full enteral feeding, days	13.5(10, 21)	17(12, 23)	0.67(0.49, 0.92)	0.01
Time to full-strength fortification, days	16(12, 23)	22.5(16, 30)	1.12(0.84, 1.49)	0.46

Median and inter-quartile range (IQR) was used to describe continuous variables; Multiple logistic regression models were used in compare the difference of feeding intolerance incidences and morbidities. Cox regression models were used in compare the difference of time to achieve full enteral feeding and time to full-strength fortification. Gestational age, sex, birth weight, age at start of HMF usage were adjusted as covariates

^*^ The number of infants in nHMF group was 94 when calculated the incidence of bronchopulmonary dysplasia, because of one infant were dead

nHMF, new human milk fortifier; cHMF, control human milk fortifier

### Safety

There were no statistically significant differences between the nHMF and cHMF group in the incidence of preterm-related morbidities, such as NEC, BPD, ROP and culture-confirmed sepsis (Table 4). During the study period, 16 AEs occurred in 13 infants in the nHMF group, including five events categorized as gastrointestinal disorder, three events as nutrition disorder, and eight events as infection disorder. In the cHMF group, 41 AEs occurred in 30 infants, including eight events as gastrointestinal disorders, eight events as nutrition disorders, and 25 events as infection-related disorders. There was no significant difference between the two groups (*P* = 0.19, not shown in Tables). Only one infant in nHMF group had severe AE: NEC (stage III) occurred after 2 days of intervention and the patient died eventually. None AEs were considered related to study product as determined by physician both in nHMF and cHMF group.

### Sensitivity analyses

In the sensitivity analyses, we compared the weight gain of all infants who completed the study (80 infants in nHMF group and 137 infants in cHMF group). As shown in the supplementary Table 2, the main outcomes, including weight gain, weight gain velocity, and change in weight-for-age z-scores of nHMF group and cHMF group were similar to the main analysis.

## Discussion

Through this non-inferiority study, compared with a historical control group, we demonstrated that supplement feeding with the new HMF in VPT infants can achieve a satisfactory and similar weight gain velocity without increasing the incidence of feeding intolerance or other major morbidities during hospitalization.

This study found that the weight gain velocities were not significantly different between the nHMF and cHMF groups from D1 to D21. However, these velocities were slower than the 17–20 g/kg/day target recommended by ESPGHAN and other studies [8, 9]. Yet, the observed weight gain velocity in our study was in line with another study conducted within Chinese population, where the standard fortification group demonstrated a weight gain velocity of 14.9 ± 4.5 g/kg/d [18]. This indicates that the nHMF can accelerate the growth of VPT infants in a similar manner as other HMFs in China. Nevertheless, some other strategies, such as individualized fortification could be implemented among Chinese VPT infants to achieve better weight gain recommended by ESPGHAN. Furthermore, we found the absolute weight gain in the nHMF group from D1 to discharge was more than that in the cHMF group while the weight gain velocity was similar to that of the control group. This might be explained by the reason that the time from D1 to discharge in the nHMF group [32 (22, 42) days] was longer than that in the cHMF group [24 (20, 33) days, *P* < 0.001, not shown in Tables]. As shown in Table 1, the time to start using HMF in the nHMF group was sooner than that of the control group, but the length of NICU stay was similar, which could explain the difference of the time from D1 to discharge between the two groups.

While this study did not observe significant differences in growth between the two groups, it was important to note that early nutrition significantly might influence the long-term development of the infant nervous system and the risk of metabolic syndrome [19, 20]. Our previous study had showed that higher protein intake during early life is associated with better body composition (with lower level of body fat mass and higher fat-free mass), which might be related with lower risk of long-term metabolic disease [21]. Compared with previous HMFs, the new powdered HMF provides higher energy, higher protein to energy ratio, and less additional carbohydrates. This suggests that feeding with the new HMF should provide more protein and less carbohydrate intake during early life, which may be associated with better body composition and long-term outcomes in later life. Regarding nutritional biomarkers, we found that the level of total cholesterol and triglyceride in nHMF group were slightly reduced, but still within the normal range, which might be related with better body composition and lower risk of cardiovascular and metabolic diseases in adulthood. However, our study did not collect data on body length, body composition, and other metabolic biomarkers, highlighting the need for more comprehensive, long-term follow-up studies to fully understand the effects of HMF on long-term metabolic outcomes.

This study supports the notion that the new HMF does not exacerbate gastrointestinal burden in VPT infants during early life. The age to achieve full enteral feeding and to full-strength fortification in the nHMF group were significantly and marginally earlier than those in cHMF group, which might be an evidence of nHMF was more tolerable. However, the age of HMF first using was also earlier in nHMF group, which indicated that the rating of feeding advancement before HMF use in nHMF group might be faster than that in the control group. This was an important confounding factor in the result explanation. This could be related to clinical protocols that allowed for a quicker progression of feeding volumes in the nHMF group. Although the age of the HMF use was adjusted as a covariate in the multifactor analyses, the influence of this factor could not be completely avoided. The formulation of the new HMF includes partially hydrolyzed whey protein and has a lower osmotic pressure. Previous studies had indicated that partially hydrolyzed proteins could serve as a beneficial alternative to intact proteins in managing common functional gastrointestinal disorders in infants [22, 23]. Furthermore, higher inflammation status of the intestine has been shown in infants with feeding intolerance than infants who without [24]. A study by Doshi et al. has shown that human milk feeding with hydrolyzed protein HMF was associated with a lower level of calprotectin (a biomarker of intestinal inflammation) in preterm infants, which might be related with better feeding tolerance [25].

We also found that the incidence of ROP in the nHMF group was slightly lower than that in the cHMF group. Human milk feeding [26] and higher energy intake [27] have been shown as protective factors for ROP. The new HMF had higher protein and calories which might contribute to reduce the incidence of ROP. However, in general, the major risk factors of ROP are gestational age, birth weight, and oxygen therapy [26]. Although the gestational age and birth weight were similar between the two groups and those were adjusted in the analyses, differences in oxygen therapy practice might exist since the control was historical. Therefore, further parallel randomized control trials are needed to confirm the effect of new HMF on ROP.

Blood nutritional indicators, such as hemoglobin, alkaline phosphatase, and BUN decreased from D0 to D21 both in nHMF and cHMF group, which is consistent with another previous study [8]. This may be related to the disease condition of preterm infants after birth. Prealbumin in the nHMF group remained stable over the period of intervention, but that in the cHMF group decreased. And we found that the decrease of BUN in nHMF group in the nHMF group was less than that in cHMF group. Both prealbumin and BUN were markers of protein intake and nitrogen balance. These results suggested that the nitrogen balance was better in nHMF group. Compare to cHMF, the nHMF provide more protein (0.36 g protein/g nHMF vs. 0.2 or 0.25 g protein/ g cHMF). This might be beneficial for the nitrogen balance of preterm infants. The current study showed that decrease of blood hemoglobin level in the nHMF group was more significant than that in the control group. Given that both groups had similar levels of hemoglobin at D21, the more pronounced decrease in the nHMF group can be explained by them starting at a higher level at D1. Although the new HMF increased the supplement of iron which might improve the hemoglobin level of preterm infants, changes of clinical management and using other iron supplementation might also play roles in this change.

This was the first study on efficacy and safety of the new HMF in China. However, some limitations exist in this study. Firstly, the data of the cHMF group were collected retrospectively from a historical infant group based on medical records. Because of time constraint, the sample size of control group was limited and other growth parameters including body length and head circumference were not available. Secondly, it is important to consider that clinical practice evolvements over time, including strategies for enteral feeding, oxygen therapy, and micronutrient supplementation. These changes could potentially lead to an overestimation of the effects of the new HMF. Thirdly, this study’s sample size was determined based solely on the primary outcome of growth velocity. Therefore, in terms of safety aspects, particularly feeding tolerance and major morbidity, the study might lack sufficient statistical power. Future studies with larger sample sizes are necessary to more definitively ascertain the impact of the new HMF on these outcomes.

## Conclusions

In conclusion, when compared to a historical control group, the new HMF demonstrated a similar efficacy in promoting the growth of preterm infants, without an increased incidence of major morbidity or feeding intolerance during hospitalization. Using the new HMF as a supplement of HM feeding is feasible in Chinese VPT/VLBW infants.

### Electronic supplementary material

Below is the link to the electronic supplementary material.

Supplementary Material 1

## Data Availability

The datasets used and/or analysed during the current study are available from the corresponding author on reasonable request.

## Abbreviations

AE: Adverse eventBPD:bronchopulmonary dysplasia
BUN: Blood urea nitrogen
CI: Confidence interval
ESPGHAN: European Society for Pediatric Gastroenterology Hepatology and Nutrition
GEE: Generalized estimating equationsHMF:Human milk fortifier
HM: Human milk
HR: Hazard ratio
IQR: Inter-quartile range LMS:Lambda-Mu-Sigma
NEC: Necrotizing enterocolitis
NICU: Neonatal intensive care unit
NIH: National Institutes of HealthOR:odds ratio
ROP: Retinopathy of prematurity
SD: Standard deviation
SGA: Small for gestational age
VPT: Very preterm
VLBW: Very low birth weight
