# Supplementary material for: Using a new human milk fortifier to optimize human milk feeding among very preterm and/or very low birth weight infants: a multicenter study in China
Source: BMC Pediatr. 2024 Jan 19;24:61. doi: 10.1186/s12887-024-04527-2 (PMC10797784; doi:10.1186/s12887-024-04527-2)
Supplement: Supplementary file 1 — Supplementary Material 1 [file 12887_2024_4527_MOESM1_ESM.docx]

**Supplementary Table 1. Nutrient compositions of human milk fortifier in the study**

| Nutritional facts | nHMF | |  | cHMF1^a^ | |  | cHMF2^a^ | |
| --- | --- | --- | --- | --- | --- | --- | --- | --- |
| Protein source | partially hydrolyzed whey protein | |  | extensively hydrolyzed whey protein | |  | non-hydrolyzed whey and casein | |
| MCT | 10% | |  | 17% | |  | 9.7% | |
|  | 1g HMF | Full-strength fortification (4g) |  | 1g HMF | Full-strength fortification (5g) |  | 0.9g HMF | Full-strength fortification (3.6g) |
| Energy (kcal) | 4.4 | 17.6 |  | 3.5 | 17.5 |  | 3.5 | 14 |
| Protein (g) | 0.36 | 1.4 |  | 0.2 | 1 |  | 0.25 | 1 |
| Fat (g) | 0.18 | 0.72 |  | 0.001 | 0.005 |  | 0.09 | 0.36 |
| Carbohydrate (g) | 0.32 | 1.28 |  | 0.67 | 3.4 |  | 0.45 | 1.8 |
| Vitamin A (ug) | 83.2 | 332.8 |  | 71.0 | 355 |  | 46.5 | 186 |
| Vitamin D (ug) | 0.88 | 3.5 |  | 0.75 | 3.8 |  | 0.75 | 3 |
| Vitamin E (mg) | 0.93 | 3.7 |  | 0.8 | 4 |  | 0.8 | 3.2 |
| Vitamin K1 (ug) | 1.9 | 7.6 |  | 1.6 | 8 |  | 2.1 | 8.4 |
| Vitamin B1 (ug) | 35 | 140 |  | 30 | 150 |  | 58.3 | 233.2 |
| Vitamin B2 (ug) | 46 | 184 |  | 40 | 200 |  | 104 | 416 |
| Vitamin B6 (ug) | 30 | 120 |  | 26 | 130 |  | 53 | 212 |
| Vitamin B12 (ug) | 0.05 | 0.2 |  | 0.02 | 0.1 |  | 0.16 | 0.64 |
| Nicotinic acid (ug) | 350 | 1400 |  | 300 | 1500 |  | 893 | 3572 |
| Folic acid (ug) | 9.4 | 37.6 |  | 8.0 | 40 |  | 5.8 | 23.2 |
| Pantothenic acid (ug) | 160 | 640 |  | 140 | 700 |  | 375 | 1500 |
| Vitamin C (mg) | 4.65 | 18.6 |  | 3.5 | 17.5 |  | 6.3 | 25.2 |
| Biotin (ug) | 0.81 | 3.24 |  | 0.70 | 3.5 |  | 6.5 | 26 |
| Inositol (mg) | 0.10 | 0.4 |  | 0.78 | 3.9 |  | 0.96 | 3.84 |
| Sodium (mg) | 9.18 | 36.7 |  | 5.2 | 26 |  | 3.8 | 15.2 |
| Potassium (mg) | 12.1 | 48.4 |  | 13.2 | 66 |  | 15.8 | 63.2 |
| Cuprum (ug) | 13 | 52 |  | 10 | 50 |  | 42.5 | 170 |
| Magnesium (mg) | 1.0 | 4 |  | 0.8 | 4 |  | 1.8 | 7.2 |
| Iron (mg) | 0.45 | 1.8 |  | 0.34 | 1.7 |  | 0.09 | 0.36 |
| Zinc (mg) | 0.24 | 0.96 |  | 0.18 | 0.9 |  | 0.25 | 1 |
| Manganese (ug) | 1.9 | 7.6 |  | 1.3 | 6.3 |  | 1.8 | 7.2 |
| Calcium (mg) | 18.9 | 75.6 |  | 15.0 | 75 |  | 29.3 | 117.2 |
| Phosphorus (mg) | 11.0 | 44 |  | 9.0 | 45 |  | 16.8 | 67.2 |
| Chlorine (mg) | 8.0 | 32 |  | 4.6 | 23 |  | 9.5 | 38 |
| Selenium (ug) | 0.65 | 2.6 |  | 0.5 | 2.5 |  | 0.13 | 0.52 |

^a^ Two kinds of HMF were used during the study periods of control group.

nHMF, new human milk fortifier; cHMF, control human milk fortifier; MCT, medium chain triglycerides

**Supplementary Table 2. Weight gain velocity and change of body weight z-score among infants in nHMF group and cHMF group**

| Weight or weight gain | nHMF group (n=80) | cHMF group (n=137) | P-Value |
| --- | --- | --- | --- |
| Weight |  |  |  |
| D1, kg | 1.51±0.25 | 1.57 ± 0.25 | 0.10 |
| D14, kg | 1.84 ± 0.31 | 1.90 ± 0.32 | 0.22 |
| D21, kg | 2.01 ± 0.34 | 2.11 ± 0.35 | 0.06 |
| Discharge, kg | 2.12 ± 0.35 | 2.28 ± 0.45 | 0.13 |
| Weight for age z-score |  |  |  |
| D1 | -0.87 ± 0.64 | -1.07 ± 0.75 | 0.06 |
| D14 | -1.10 ± 0.67 | -1.29 ± 0.88 | 0.14 |
| D21 | -1.15 ± 0.73 | -1.29 ± 0.93 | 0.45 |
| Discharge | -1.20 ± 1.00 | -1.24 ± 0.88 | 0.79 |
| Weight gain from D1 to D21 |  |  |  |
| Absolute weight gain, g | 527 ± 159 | 541 ± 175 | 0.67 |
| Weight gain velocity, g/kg/d | 14.1 ± 3.4 | 14.1 ± 3.8 | 0.89 |
| Change of z-score | -0.26 ± 0.40 | -0.19 ± 0.44 | 0.34 |
| Weight gain from D1 to discharge |  |  |  |
| Absolute weight gain, g | 916 ± 481 | 727 ± 376 | 0.001 |
| Weight gain velocity, g/kg/d | 13.7 ± 4.3 | 14.3 ± 6.5 | 0.67 |
| Chang of z-score | -0.32± 0.74 | -0.18 ± 0.45 | 0.10 |
| Weight gain from D1 to D14 |  |  |  |
| Absolute weight gain, g | 336 ± 121 | 333 ± 138 | 0.67 |
| Weight gain velocity, g/kg/d | 14.3 ± 4.3 | 13.7 ± 4.9 | 0.29 |
| Change of z-score | -0.21 ± 0.32 | -0.19 ± 0.35 | 0.86 |

Mean and standard deviation (SD) was used to describe continuous variables.

D1, D14, D21, the 1^st^, 14^th^, and 21^st^ day after full-strength fortification; nHMF, new human milk fortifier; cHMF, control human milk fortifier.
